# Up-Regulation of TREK-2 Potassium Channels in Cultured Astrocytes Requires *De Novo* Protein Synthesis: Relevance to Localization of TREK-2 Channels in Astrocytes after Transient Cerebral Ischemia

**DOI:** 10.1371/journal.pone.0125195

**Published:** 2015-04-17

**Authors:** Aixa F. Rivera-Pagán, David E. Rivera-Aponte, Katya V. Melnik-Martínez, Astrid Zayas-Santiago, Lilia Y. Kucheryavykh, Antonio H. Martins, Luis A. Cubano, Serguei N. Skatchkov, Misty J. Eaton

**Affiliations:** 1 Department of Biochemistry, Universidad Central del Caribe, Bayamón, Puerto Rico, United States of America; 2 Departments of Anatomy and Cell Biology, Universidad Central del Caribe, Bayamón, Puerto Rico, United States of America; 3 Department of Physiology, Universidad Central del Caribe, Bayamón, Puerto Rico, United States of America; National University of Singapore, SINGAPORE

## Abstract

Excitotoxicity due to glutamate receptor over-activation is one of the key mediators of neuronal death after an ischemic insult. Therefore, a major function of astrocytes is to maintain low extracellular levels of glutamate. The ability of astrocytic glutamate transporters to regulate the extracellular glutamate concentration depends upon the hyperpolarized membrane potential of astrocytes conferred by the presence of K^+^ channels in their membranes. We have previously shown that TREK-2 potassium channels in cultured astrocytes are up-regulated by ischemia and may support glutamate clearance by astrocytes during ischemia. Thus, herein we determine the mechanism leading to this up-regulation and assess the localization of TREK-2 channels in astrocytes after transient middle cerebral artery occlusion. By using a cell surface biotinylation assay we confirmed that functional TREK-2 protein is up-regulated in the astrocytic membrane after ischemic conditions. Using real time RT-PCR, we determined that the levels of TREK-2 mRNA were not increased in response to ischemic conditions. By using Western blot and a variety of protein synthesis inhibitors, we demonstrated that the increase of TREK-2 protein expression requires De novo protein synthesis, while protein degradation pathways do not contribute to TREK-2 up-regulation after ischemic conditions. Immunohistochemical studies revealed TREK-2 localization in astrocytes together with increased expression of the selective glial marker, glial fibrillary acidic protein, in brain 24 hours after transient middle cerebral occlusion. Our data indicate that functional TREK-2 channels are up-regulated in the astrocytic membrane during ischemia through a mechanism requiring De novo protein synthesis. This study provides important information about the mechanisms underlying TREK-2 regulation, which has profound implications in neurological diseases such as ischemia where astrocytes play an important role.

## Introduction

Astrocytes, the most numerous cells in the human brainstem and cortex [1], are essential for neuronal viability, in part, by maintaining extracellular homeostasis. Maintaining the hyperpolarized astrocytic resting membrane potential plays a fundamental role in regulating the glial contribution to buffering of potentially toxic neurotransmitters, such as glutamate that is released during ischemia. The hyperpolarized membrane potential of astrocytes is primarily due to potassium channels in their membranes [2–5]. Although Kir4.1 potassium channels, a member of the family of inward rectifying K^+^ (Kir) channels, are predominantly responsible for maintaining the hyperpolarized membrane potential of astrocytes and in extracellular K^+^ buffering under normal conditions [2–5], members of the tandem pore family of K^+^ channels (such as TREK-1 and TREK-2 channels) have also been ascribed a role in this process, particularly during conditions of stress, such as during ischemia [6–10]

TREK-2 tandem-pore domain channels are functionally expressed in astrocytes [6,11–12] and are targets of many physiological stimuli; TREK-2 channels can be activated by polyunsaturated fatty acids, intracellular acidosis, temperature and by mechanical stretch [7,13]. During ischemia, activation of phospholipases promotes liberation and accumulation of arachidonic acid [14], the intracellular pH of astrocytes becomes acidic and astrocytes swell [15–16]. All of these changes would cause activation of TREK-2 channels and therefore, it has been hypothesized that TREK-2 in astrocytes may help maintain extracellular K^+^ and glutamate concentrations low during pathological events such as anoxia, ischemia, hypoxia, hypoglycemia and/or spreading depression [6,8]. Indeed, it has recently been shown that TREK-2 potassium channels in astrocytes support glutamate clearance during ischemic conditions [7] and are functionally up-regulated by ischemia [7,17–18]. The purpose of the present study was to determine the mechanism leading to up-regulation of functional TREK-2 channels in astrocytes during an ischemic insult. There are a number of ways that TREK-2 protein levels can be up-regulated after ischemia. The first is by increased transcription followed by increased translation of the protein. Additionally, post-transcriptional mechanisms could be involved in TREK-2 up-regulation. These include: 1) regulation of translation by factors binding to the untranslated region (UTR) of mRNA [19], 2) liberation of TREK-2 mRNA from production bodies or p-bodies [20], 3) increased insertion of channel protein into the plasma membrane [21], and/or 4) decreased degradation of protein [22]. All of these processes would result in increased TREK-2 protein within the astrocyte. In the present study, we examined whether the up-regulation of TREK-2 protein levels is due to changes in transcription, translation and/or degradation of astrocytic TREK-2 during ischemia. In addition, we provide *in vivo* evidence for up-regulation of astrocytic TREK-2 channels after middle cerebral artery occlusion (MCAO) using immunocytochemistry and immunoblotting.

## Methods

### Animals

Experiments were carried out in accordance with a protocol approved by the Universidad Central del Caribe Institutional Animal Care and Use Committee (UCC-IACUC). Adequate measures were taken to minimize pain or discomfort to experimental animals. Adult and 1–2 postnatal days Sprague-Dawley rats were used.

### Materials used

Cycloheximide, Emetine, Puromycin and Chloroquine were purchased from Sigma Chemical Co. (St. Louis, MO). Calpeptin and MG132 were purchase from Tocris Bioscience (Minneapolis, MN).

### Astrocyte primary cultures

Primary astrocyte cultures were prepared from neocortex of 1–2 day old rats as previously described [3]. Briefly, brains were removed after decapitation and the meninges stripped away to minimize fibroblast contamination. The forebrain cortices were collected and dissociated using the stomacher blender method. The cell suspension was then allowed to filter by gravity only through a #60 sieve and then through a #100 sieve. After centrifugation, the cells were suspended in Dulbecco’s Modified Eagle Medium (DMEM) containing 25mM glucose, 2mM glutamine, 1mM pyruvate and 10% fetal calf serum, and plated on uncoated 75 cm^2^ flasks at a density of 300,000 cells/cm^2^. At confluence (about 12–14 days), the mixed glial cultures were treated with 50mM leucine methylester (pH 7.4) for 60 min to kill microglia. Cultures were then allowed to recover for at least one day in growth medium prior to reseeding. Astrocytes were dissociated by trypsinization and reseeded onto appropriate plates for the experiments.

### Simulated ischemia

Astrocytes were exposed to hypoxia/hypoglycemia for 24 hours to simulate ischemia in vitro. Astrocytes were plated in petri dishes and to achieve hypoxia/hypoglycemic conditions the medium from cultures was removed, cells were gently rinsed and a bicarbonate-buffered balanced salt solution (BBSS) containing: 127mM NaCl, 3mM KCl, 19.5mM NaHCO_3_, 1.5mM NaH_2_PO4, 1.4mM MgSO_4_, 1mM CaCl and 2.5mM D-glucose and previously gassed for 5 minutes with 95% N_2_ and 5%CO_2_ was applied to the cells. Afterwards, cells were placed in a chamber flooded with 95% N_2_ and 5% CO_2_ and incubated at 37°C for 24 hours. Control cells were incubated in a BBSS solution containing 25mM D-glucose and incubated at 37°C for 24 hours under normoxic conditions. We have previously shown using electrophysiology that astrocytes exposed to these conditions are viable [7].

### SDS-PAGE and Western blotting analysis

Cultured astrocytes were pelleted and resuspended in lysis buffer (pH 7.5) containing: (in mM) Tris-HCl 20, NaCl 150, EDTA 1.0, EGTA 1.0, PMSF 1.0, 1% Triton X-100, and an additional mixture of peptide inhibitors (leupeptin, bestatin, pepstatin, and aprotinin). Brain slices were lysed using a homogenizer in RIPA buffer (TRIS-HCl 1.5M pH8.8, 2%Triton, NaCl 150mM and 10%SDS). Total protein of homogenates were determined with the DC protein assay (Bio-Rad), followed by addition of an appropriate volume of Urea sample buffer (62mM Tris/HCl pH 6.8, 4% SDS, 8M Urea, 20mM EDTA, 5% β-Mercaptoethanol, 0.015% Bromophenol Blue) to load 15 μg of protein per lane. Next, samples were boiled in a water bath at 95°C for 10 minutes, spun to pellet debris, and immediately run on 10% SDS-polyacrylamide gels. Western blotting was performed as previously described [7] using rabbit polyclonal antibodies against TREK-2 (1:200; Santa Cruz Biotechnology, SC98688 or 1:1000; Millipore, AB5933). Final detection was performed with enhanced chemiluminescence methodology (SuperSignal West Dura Extended Duration Substrate; Pierce, Rockford, IL) as described by the manufacturer, and the intensity of the signal was measured in a gel documentation system (Versa Doc Model 1000, Bio Rad). In all cases, intensity of the chemiluminescence signal was corrected for minor differences in protein content after densitometry analysis of the India ink stained membrane. TREK-2 was detected as a band of around 60kDa which is consistent with the predicted molecular weight of 59.6kDa. In some experiments, a positive control (Origene, cat. no. LY412052) was used to validate the identity of the band

### Cell surface biotinylation

Cultured astrocytes were plated in 100-mm dishes at 90% confluency. After exposing the cells to ischemic conditions for 24 hours, cell surface protein biotinylation and extraction of cytosolic and surface membrane fractions were performed, using the Pierce cell surface protein isolation kit (Thermo Scientific). Briefly, the cells were washed in ice-cold phosphate buffered solution (PBS) and incubated in Sulfo-NHS-SS-Biotin solution (0.25 mg/ml, dissolved in PBS pH 7.4) at 4°C for 30 min. At this time, the reaction was quenched, and the cells were collected and incubated for 30 min in lysis buffer; during the incubation, the reaction was sonicated to help disrupt the cells. The lysate was centrifuged, and the supernatant was added to columns containing immobilized neutravidin gel for biotin binding and incubated for 1 h at room temperature. The columns were centrifuged (1000 X *g*, 1 min), and the flow through was collected as the cytosolic protein fraction and mixed with SDS-PAGE sample buffer (62.5mM Tris/HCl, pH 6.8, 1% SDS, 10% glycerol) containing 5% mercaptoethanol. Subsequent to washing with a buffer containing 1% protease inhibitor mixture (Sigma), 400 μl of SDS-PAGE buffer with 50 mM DTT was added to the columns. After 1 h of incubation at room temperature, the columns were centrifuged, and the flow through was recovered as the cell surface membrane protein fraction. Trace amounts of bromophenol blue were added to the membrane and cytoplasmic fractions before analysis by Western blot.

### Real time RT-PCR

TREK-2 mRNA levels were assessed by real time RT-PCR analysis for the Kcnk10 (TREK-2) RNA transcript. Total RNA (in 50μl of water) was isolated using an RNeasy Kit (Qiagen). 10μl of each RNA sample were used for the determination of mRNA using a LightCycler (Roche Diagnostics, Indianapolis, IN) and a LightCycler Amplification kit SYBR green 1 (Roche Diagnostics, cat. no. 12015137001). Amplification reactions (20μl) contained 3mM MgCl_2_, 0.5μM of each primer (validated sense and antisense primers for the Kcnk10 gene; Qiagen, cat. no. QT00193823) and 2μl of 10X LightCycler FastStart DNA Master SYBR Green I (Roche Diagnostics). PCR amplifications were carried out in glass capillary tubes (Roche Diagnostics). Reverse transcription was performed at 55°C for 600 s, and amplification began with a 30 s denaturation step at 95°C followed by 45 cycles of annealing at 55°C for 10 s and extension at 72°C for 13 s. We optimized the results by performing a standard curve for the mRNA using the LightCycler Control Kit DNA (Roche Diagnostics, cat. no. 12158833001). The data were analyzed using LightCycler Software (Roche Molecular Biochemicals).

### Middle cerebral artery occlusion

Adult Sprague-Dawley rats (250–300 g) were anesthetized with 5% isoflurane for induction and 2% for maintenance and subjected to a transient Middle Cerebral Artery Occlusion (tMCAO). Briefly, a ventral midline incision was made in the neck and the left common carotid artery exposed, the external carotid was isolated and permanently ligated as well as the pterygopalatine artery. The occipital and thyroid arteries were electrocoagulated. The blood flow was stopped by using artery clips and the external carotid was transected. A piece of monofilament suture (Doccol) was inserted into the lumen of the stump and advanced into the internal carotid artery until resistance was found, showing that it is in the middle cerebral artery [23]. Restoration of blood flow was achieved 60 minutes later by withdrawal of the intraluminal monofilament. After 24 hours of reperfusion, animals were anesthetized with isoflurane, decapitated and brains were removed and sectioned by using a rat brain matrix to obtain coronal slices/blocks at 1.0 mm intervals. Alternate sections obtained using the brain matrix were used for triphenyltetrazolium chloride (TTC) staining (5% TTC in PBS, pH 7.4 for 15–20 minutes) and either analysis by Western blot or immunohistochemistry.

### Immunohistochemistry

The brains sections adjacent to the TTC stained sections were fixed in 4% paraformaldehyde in PBS (pH 7.4) for 2 hours at 4°C and subsequently sectioned to 50μm using a vibroslicer (Leica VT1000S, Leica, Germany). The 50μm sections were permeabilized in PBS containing 0.3% Triton X-100, and 3% normal goat serum for 30 min. The brain slices were co-incubated in polyclonal anti-TREK-2 antibody (1/200; Santa Cruz Biotechnology) and monoclonal mouse anti-glial fibrillary acidic protein (GFAP) antibody tagged with Cy3 (1/1000; Sigma-Aldrich, C9205) for 24 hrs at 4°C. After washing, slices were incubated in a mixture of biotinylated goat anti-rabbit IgG (1:5000) for 24 hours at 4°C. The slices were subsequently incubated in Avidin-FITC in PBS for 2 hours at room temperature. After washing in PBS, the slices were placed on Superfrost plus microscope slides and coverslipped. As a control sections were processed as described above with omission of the primary antibody.

## Results

### TREK-2 expression is increased in the astrocytic membrane after ischemia

**Fig 1:**
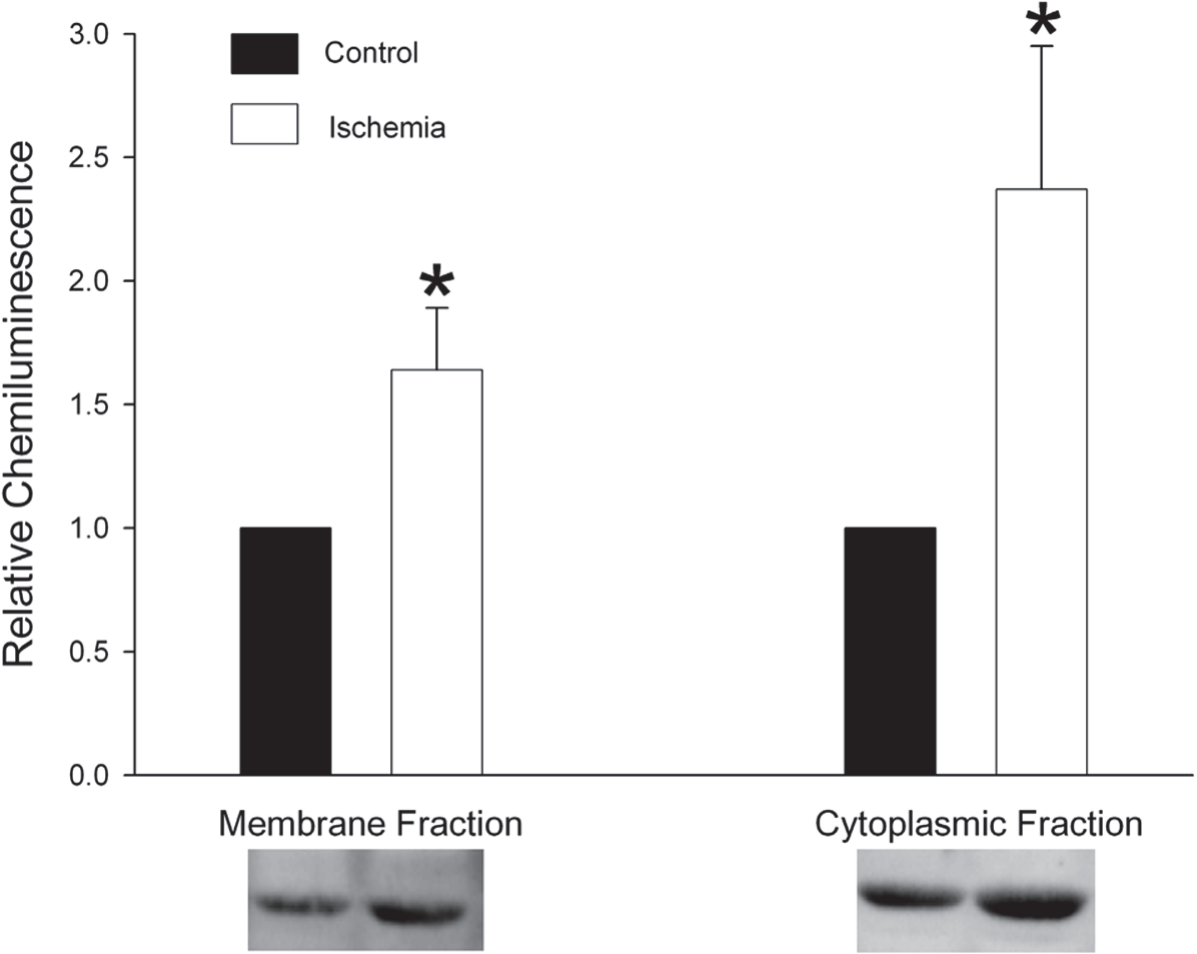
TREK-2 expression is increased in the astrocytic membrane and cytoplasm after ischemia. Cortical astrocytes in culture were exposed to hypoxic/hypoglycemic conditions for 24 hours and then processed using a cell surface biotinylation assay. The graph displays the quantification of the relative chemiluminescence intensity ± standard error of the mean (SEM) of TREK-2 protein in membrane and cytoplasmic fractions obtained from control astrocytes and astrocytes subjected to ischemia (representative Western blots shown below the graph). TREK-2 was detected as a band of around 60kDa which is consistent with the predicted molecular weight of 59.6kDa. The results of 4 separate experiments using different astrocyte cultures are shown. The asterisks indicate a significant difference from control (t-test; p<0.05). Data are expressed relative to control.

TREK-2 protein and mRNA expression have been reported in cortical and hippocampal astrocytes [5,6,12] and TREK-2 protein levels together with TREK-like electrical currents are up-regulated after hypoxic/hypoglycemic conditions [7]. Therefore, we questioned if TREK-2 channel proteins are located in cytoplasmic vesicles or within the membrane of the astrocytes? This is an important issue since tandem-pore domain potassium channels maintain glial membrane potential and function [24,25] and specifically TREK-2 in astrocytes [7]. Using a cell surface biotinylation assay, we now demonstrate that TREK-2 protein levels were significantly increased in both cytoplasmic (2.36 fold ± 0.58 SEM) and membrane fractions (1.64 ± 0.25 SEM) obtained from astrocytes after 24 hours of hypoxia/hypoglycemic conditions (Fig 1). These data confirm that the increased outward current in astrocytes exposed to ischemic conditions previously reported in Kucheryavykh et al. [7] is due to a greater number of functional TREK-2 channels in the astrocytic membrane. Although the mechanism involved in TREK-2 channel up-regulation is still unknown.

### TREK-2 mRNA is not increased after ischemia

In response to ischemia, there is increased transcription of some selective genes in astrocytes such as the one encoding for the intermediate filament glial fibrillary acidic protein [26]. In this experiment, we tested the possibility that transcription of the gene encoding TREK-2 is similarly up-regulated after ischemia, which, in turn, could account for increased synthesis of TREK-2 protein. We used real time RT-PCR to assess changes in TREK-2 mRNA levels in control astrocytes and in astrocytes exposed to hypoxia/hypoglycemia. Surprisingly, we found that TREK-2 mRNA is not increased (0.94 arbitrary units ± 0.10 SEM) after 24 hours of ischemic-like conditions relative to control. These results were obtained from 3 separate experiments using different astrocyte cultures. These data suggest that TREK-2 channel protein levels are up-regulated during ischemia through a mechanism independent from De novo RNA synthesis.

### *De novo* protein synthesis is involved in up-regulation of TREK-2 in response to ischemia

**Fig 2:**
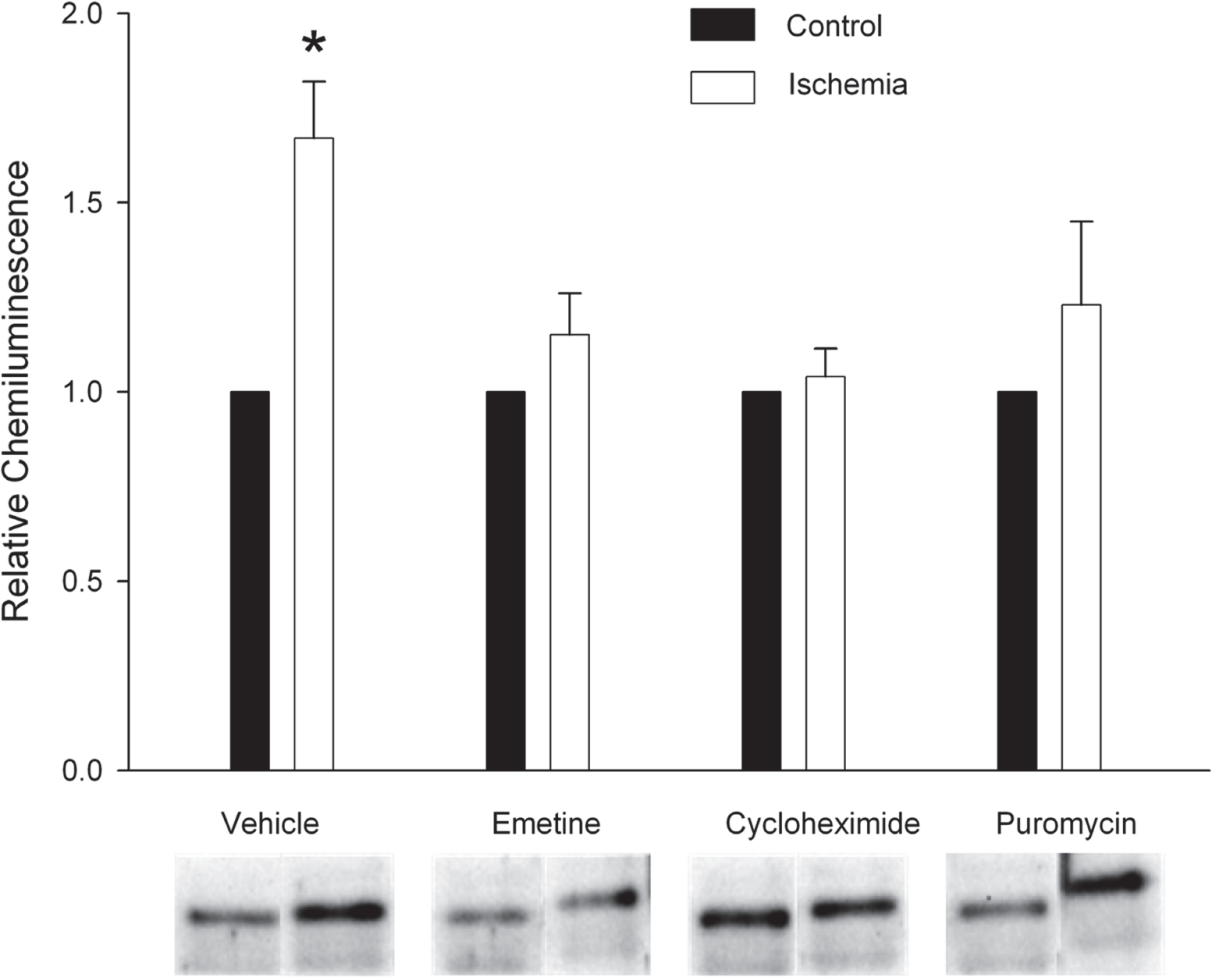
TREK-2 up-regulation in response to ischemia is due to De novo protein synthesis. Cortical astrocytes were treated with different protein synthesis inhibitors (1μg/mL cycloheximide, 300nM emetine, 5μg/mL puromycin) or without treatment (vehicle) for 24 hours. The cells were then exposed to control or hypoxia/hypoglycemic conditions for 24 hours still in the presence of the inhibitors. Afterwards, cells were harvested and TREK-2 protein levels determined by Western blot. The graph summarizes the effect of three different protein synthesis inhibitors on TREK-2 up-regulation during ischemia. The data are expressed as relative chemiluminescence intensity ± SEM. The results of 6 separate experiments using different astrocyte cultures are shown. The asterisk indicates significant difference from the corresponding control (ANOVA followed by Tukey’s test; p<0.05).

Increases in protein synthesis may occur together with or independent from changes in mRNA synthesis [27–29]. Since we observed no effect of hypoxia/hypoglycemic conditions on TREK-2 mRNA levels, we examined the possibility that up-regulation of TREK-2 channels is due to De novo protein synthesis. If increased TREK-2 protein levels require De novo synthesis of TREK-2, then inhibitors of protein synthesis should obviate differences in TREK-2 protein levels between control astrocytes and astrocytes exposed to hypoxia/hypoglycemia. To assess protein synthesis inhibition, astrocytes were treated with different protein synthesis inhibitors (300nM emetine, 1μg/mL cycloheximide, or 5μg/mL puromycin) and then exposed to control or hypoxia/hypoglycemic conditions for 24 hours. Afterwards, cells were harvested and TREK-2 protein levels determined by Western blot. We found that TREK-2 up-regulation was blocked by pharmacological blockade using protein synthesis inhibitors (Fig 2). In control cells, ischemia increased TREK-2 levels measured as relative chemiluminescence by 1.76 fold ± 0.15 SEM. The relative chemiluminescence determined for the ischemic treatment in the presence of protein synthesis inhibitors was 1.15 ± 0.10, 1.04 ±. 074, 1.23 ± 0.22 SEM for emetine, cycloheximide and puromycin, respectively. This indicates that up-regulation of TREK-2 protein requires De novo protein synthesis. An additional alternative possibility is that ischemia could decrease the rate of TREK-2 protein degradation, which would also result in an increase in protein content. Therefore, we tested this possibility.

### TREK-2 protein degradation is not altered after ischemia

**Fig 3:**
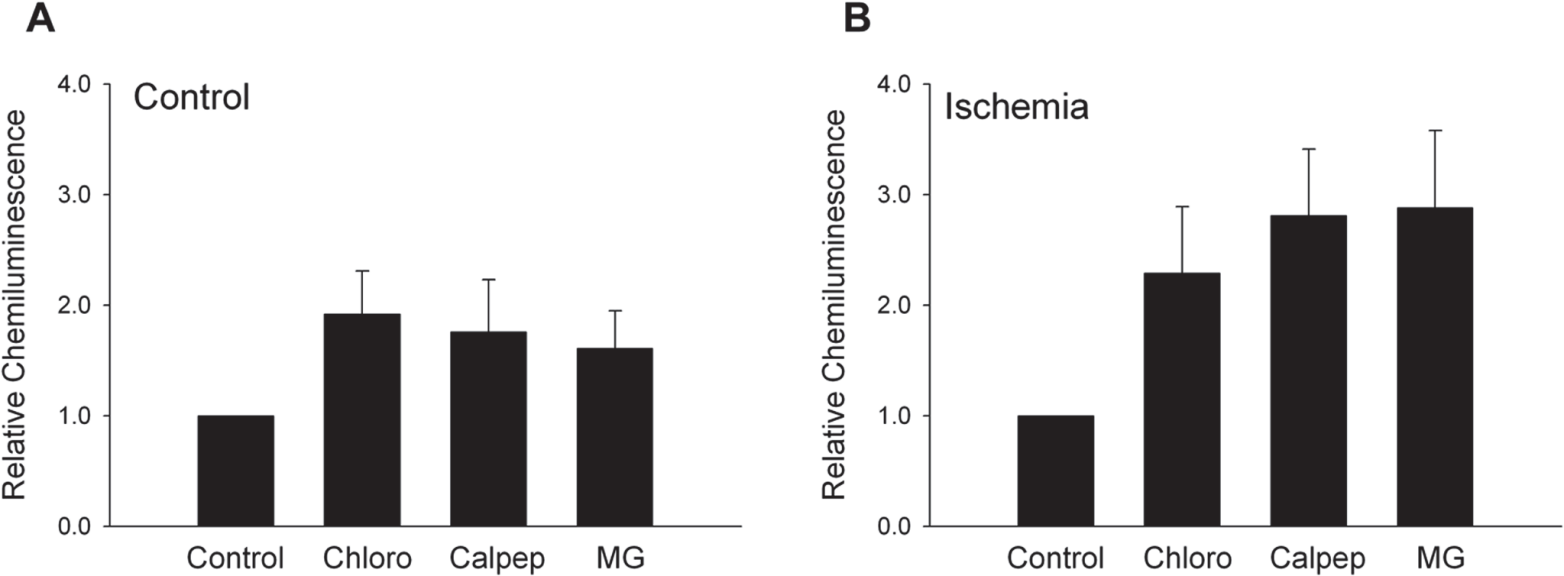
TREK-2 protein degradation pathway(s) are not altered after ischemia. Cortical astrocyte in cultures exposed to (A) control or (B) hypoxia/hypoglycemic conditions for 24 hours were treated with either 10 μM chloroquine, 50 μM calpeptin or 10 μM MG132 for 8 hours. Astrocytes were initially exposed to hypoxia/hypoglycemic conditions for 16 hours. The cells were then treated with the degradative pathway inhibitors or without treatment and returned to hypoxia/hypoglycemic conditions for an additional 8 hours. TREK-2 expression in astrocytes was determined by Western blot. The results of 3 separate experiments using different astrocyte cultures are shown. Data are expressed relative to control.

Regulation of protein degradation plays an active role in determining levels of proteins in a cell [30–32]. There are 3 major degradative pathways in mammalian cells, the Ubiquitin proteasome pathway, the Lysosomal pathway and the Calpain pathway. Thus, we next determined the normal degradation pathway(s) for TREK-2 within astrocytes during control or hypoxia/hypoglycemic conditions. Astrocytes exposed to control or hypoxia/hypoglycemic conditions for 24 hours and were incubated for the last 8 hours with either a proteasome inhibitor (10μM MG132), a lysosomal inhibitor (10μM chloroquine) or a calpain inhibitor (50μM calpeptin) after which the cells were harvested for Western blot. Control cells were run in parallel and incubated for 8 hours after addition of equal volume of the BBSS vehicle used for inhibitors. Inhibition of the pathway/pathways involved in TREK-2 degradation will result in increased TREK-2 protein within the cell. Fig 3A shows that the ubiquitin, lysosomal and calpain degradation pathways all seem to be involved in the regulation of TREK-2 protein degradation during control conditions, though these data could not be confirmed statistically. The relative chemiluminescence as compared to control was 2.09 ± 0.40, 1.92 ± 0.78 and 1.62 ± 0.62 SEM for chloroquine, calpeptin and MG132, respectively. When we examined if protein degradation plays a role during hypoxic/hypoglycemic conditions we found that the contribution of the different protein degradation pathways was not altered during ischemia, i.e., all three pathways were still being utilized for TREK-2 protein degradation (Fig 3B). The relative chemiluminescence as compared to control was 2.04 ± 0.57, 2.31 ± 0.71 and 2.26 ± 0.87 SEM for chloroquine, calpeptin and MG132, respectively.

### Localization of TREK-2 channels in astrocytes after transient middle cerebral artery occlusion

**Fig 4:**
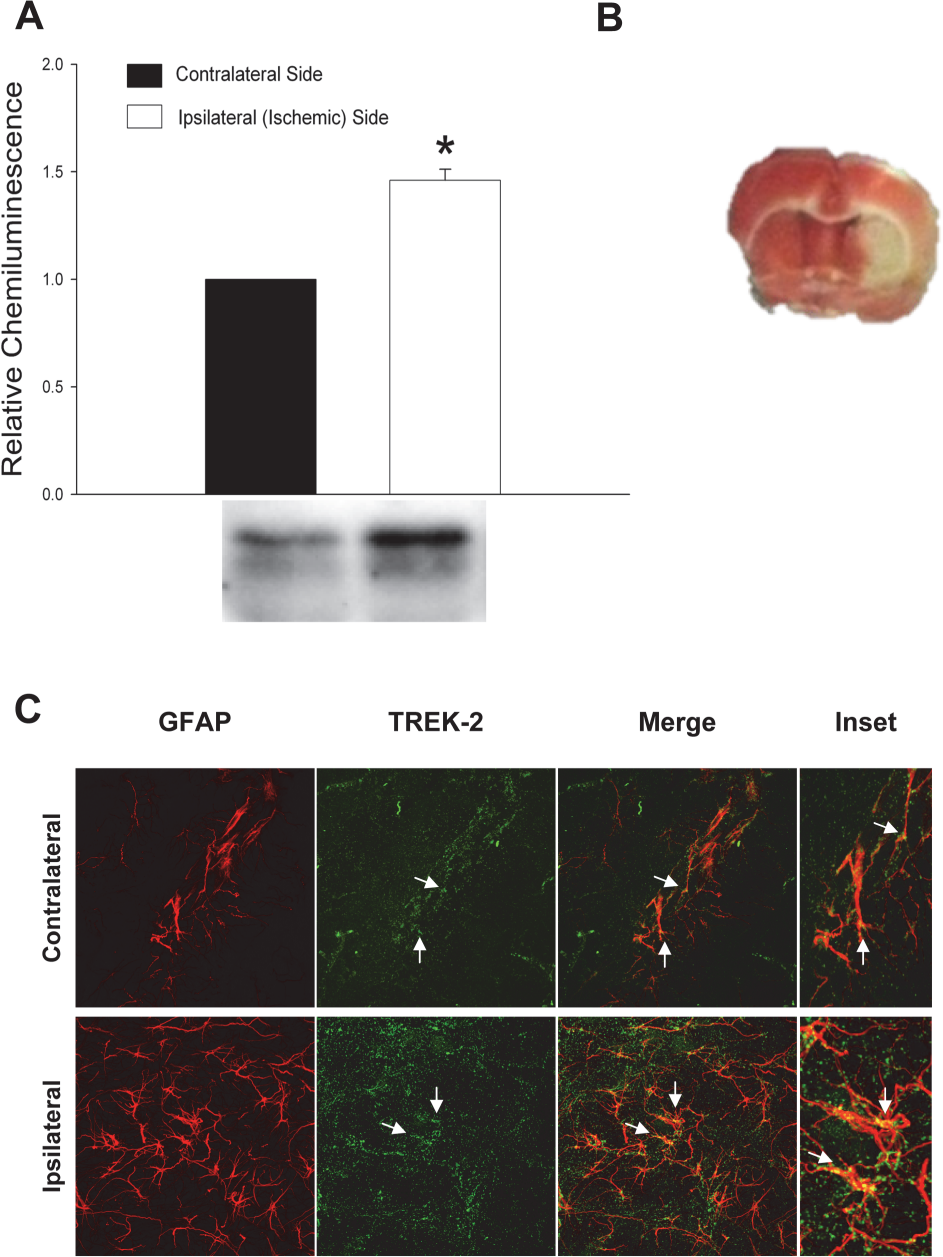
TREK-2 protein is up-regulated in vivo in astrocytes after tMCAO. (**A**) Expression changes of TREK-2 channel protein in brain after tMCAO. The results of 4 separate tMCAO experiments are shown. The asterisk indicates a significant difference from contralateral side (control) ± SEM (t-test; p<0.05). (**B**) TTC staining delineates a clearly detectable lesion at the infarct core (left side) at 24 hours after 60 minutes of tMCAO. (**C**) Immunostaining for TREK-2 (green labeling) and GFAP (red labeling) in hippocampus after tMCAO. Representative images show a qualitative increase of TREK-2 levels in hippocampus (**C**) on the ipsilateral (lesion) side of the brain. White arrows point to astrocytic processes and endfeet. Insets show higher magnification of the merged image to highlight colocalization between GFAP and TREK-2 channels in astrocytes.

Using Western blot, we assessed the expression changes of TREK-2 channels in rat brain after transient middle cerebral artery occlusion (tMCAO). Fig 4A shows that TREK-2 protein was up-regulated (1.46 fold ± 0.052 SEM) on the ipsilateral side of the brain receiving the ischemic insult (left side) as compared with the contralateral side (right side). These data correlate with what has been previously reported by Li et al. [17], where they showed that TREK-2 expression was significantly increased in cortex and hippocampus 24 hours after tMCAO. Nevertheless, the authors concluded that TREK-2 up-regulation occurred in cortical and hippocampal neurons, but did not assess the effect of ischemia on astrocytes in the brain. Our next series of experiments directly addressed this question.

We previously demonstrated that TREK-2 channel protein is increased in cultured astrocytes after in vitro experimental ischemia [7]. Therefore, to demonstrate up-regulation of astrocytic TREK-2 channels in vivo we determined the immunohistochemical localization of TREK-2 channels in astrocytes after tMCAO. First, we corroborated that the tMCAO was effective by using triphenyltetrazolium chloride (TTC). TTC staining revealed the white/pale area, which corresponds to the infarcted area (left side) in the brain 24 hours after tMCAO (Fig 4B). Next, we performed immunohistochemical staining from brain sections adjacent to the ones used for TTC staining. We examined GFAP positive glial cells at the border of the infarction area and in corresponding areas on the contralateral side of the brain and determined qualitatively if TREK-2 co-localizes with GFAP; a marker of reactive astrocytes. We observed an increase in GFAP and TREK-2 expression on the lesioned side of the brain in hippocampus (Fig 4C) and in the cortex (S1 Fig) above the striatum where the lesion is located as compared with the contralateral side. Furthermore, double immunofluorescence labeling revealed that TREK-2 channels were localized in astrocytes (Fig 4C and S1 Fig).

## Discussion

Despite the increasing evidence that TREK-2 channels might be playing an important role in neuroprotection during pathological conditions [6–8,17–18,33], the mechanism(s) underlying TREK-2 up-regulation during ischemia are not well understood. In the present work, we confirm that the increase in potassium currents observed in astrocytes exposed to ischemia previously reported by our laboratory [7] is indeed due to an increase of TREK-2 protein within the astrocytic membrane. These data indicate that the increase in the numbers of TREK-2 channels in the astrocytic membrane contribute to maintaining the hyperpolarized membrane potential of astrocytes during ischemia. Thus, TREK-2 may help maintain extracellular glutamate and K^+^ homeostasis during pathological events such as anoxia, ischemia, hypoxia, hypoglycemia and/or spreading depression [6–7,17]. Increased astrocytic expression of TREK-2 channels would be particularly beneficial in the areas of brain surrounding the ischemic core which is generally considered the salvageable regions after a stroke. In these regions, the elevations of extracellular potassium are transient [34] probably due to uptake of extracellular K^+^ through the Na^+^/K^+^ pump [35]

The current study provides the first evidence of the mechanism involved in TREK-2 regulation during ischemia. Using real time RT-PCR, we found that TREK-2 mRNA is not increased after 24 hours of ischemic-like conditions. This result was not entirely unexpected because Xu et al. [18] reported that TREK-2 mRNA levels did not change in rat cortex and hippocampus after 3 and 30 days of permanent bilateral carotid artery ligation. Furthermore, we previously observed significant elevation in TREK-2 protein very rapidly within two hours of the onset of ischemic conditions [7], suggesting that some mechanism other than increased transcription may be involved. It is noteworthy that Li and colleagues [17] reported an increase of TREK-2 mRNA levels after 24 hours of tMCAO. Considering the discrepancies between our and Xu et al. [18] results with those of Li et al. [17], we suggest the following two possibilities: 1) the mRNA response depends on the nature of the ischemic insult and/or 2) since our study was performed in isolated astrocytes and the Li et al. [17] study was performed in whole brain, this difference in mRNA response could be attributed to a difference between the mechanisms of neurons and astrocytes for up-regulating TREK-2 in response to ischemia. Further studies need to be performed to corroborate these possibilities.

Our findings show that up-regulation of TREK-2 protein in response to ischemia was blocked by protein synthesis inhibitors. Moreover, we found no evidence that protein degradation pathway(s) play an active role in TREK-2 protein regulation. Although some increase in protein levels were observed with the protein degradation inhibitors in the hypoxic situation, this increase could be attributed to the higher levels of TREK-2 in the cells. Taken together these data indicate that up-regulation of TREK-2 in response to ischemia requires De novo protein synthesis.

Based on these finding we speculate that processing bodies (P bodies) formation and/or microRNAs may be post-transcriptional regulators. P bodies are aggregates in the cytoplasm that associate with mRNAs to repress protein synthesis by either degrading the mRNA or storing it to later release it for protein translation [36–37]. Liu and colleagues [38] found that mRNAs can be repressed in P bodies in a miRNA-dependent manner. Therefore, we propose that TREK-2 mRNA could be stored in P bodies by miRNA during normal conditions and then released to resume translation during pathophysiological conditions such as ischemia. This assumption opens a new area of study and requires further research, which is beyond the scope of the current study.

Astrocytic TREK-2 channels have been previously studied in vitro [7,12,33] however, studies of TREK-2 channels in astrocytes in rat ischemic models have not yet been performed. This study provides the first histological evidence of TREK-2 channel localization in astrocytes in rat brain. Double labeling shows that GFAP positive astrocytes express TREK-2 channels.

Furthermore, TREK-2 immunolabel is increased in astrocytes in the lesioned side of the brain 24 hours after tMCAO. GFAP is a classical marker for reactive astrocytes and its expression is up-regulated in response to ischemic brain injury [26]. These findings correlate with our present study and our previous study where we showed that TREK-2 channel expression was increased in cultured cortical astrocytes in response to ischemic conditions [7]. Reactive astrocytes appear to have a neuroprotective role in ischemic brain [39] perhaps TREK-2 channels contribute to this neuroprotection by maintaining astrocytic homeostatic functions, such as glutamate clearance [7]. Taken together, our findings provide a better understanding of the mechanism that results in functional up-regulation of TREK-2 channels in astrocytes during an ischemic insult. Astrocytic TREK-2 channels may play an important role in neuroprotection during neurological diseases such as ischemia due to stroke, thus, understanding how these channels are regulated in astrocytes may lead to the development of new therapeutic agents to prevent neurotoxicity during pathological events such as ischemia.

## Supporting Information

S1 Fig Localization of TREK-2 channels in cortical astrocytes in brain. Immunostaining for TREK-2 (green labeling) and GFAP (red labeling) in cortex after tMCAO. Representative images show a qualitative increase of TREK-2 levels in cortex on the ipsilateral (lesion) side of the brain. White arrows point to astrocytic processes and endfeet. Insets show higher magnification of the merged image to highlight colocalization between GFAP and TREK-2 channels in astrocytes.
(TIF)
