# Supplementary figures and images for: Up-Regulation of TREK-2 Potassium Channels in Cultured Astrocytes Requires De Novo Protein Synthesis: Relevance to Localization of TREK-2 Channels in Astrocytes after Transient Cerebral Ischemia
Source: PLoS One. 2015 Apr 17;10(4):e0125195. doi: 10.1371/journal.pone.0125195 (PMC4401746; doi:10.1371/journal.pone.0125195)

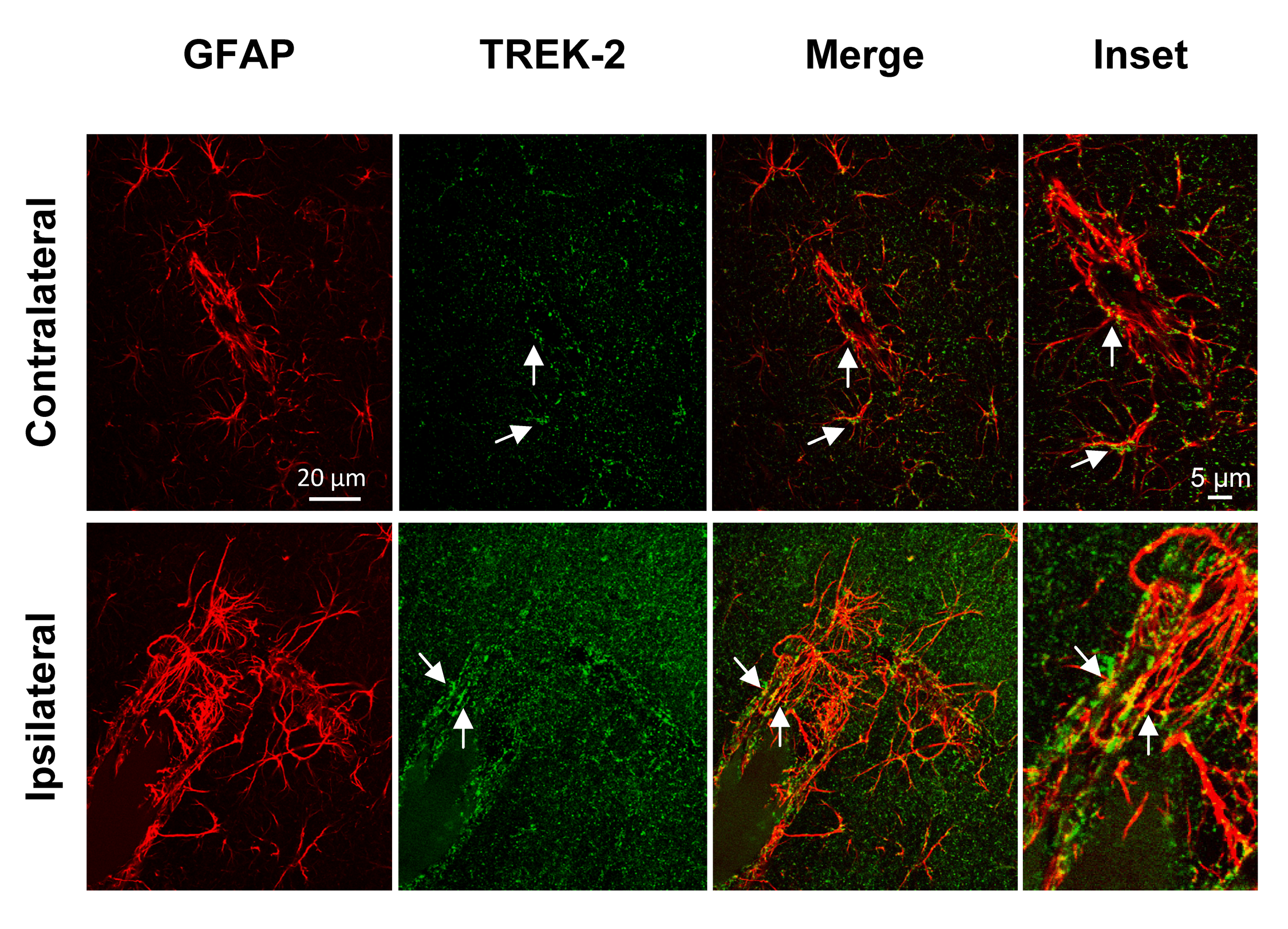

Supplement: S1 Fig — Immunostaining for TREK-2 (green labeling) and GFAP (red labeling) in cortex after tMCAO. Representative images show a qualitative increase of TREK-2 levels in cortex on the ipsilateral (lesion) side of the brain. White arrows point to astrocytic processes and endfeet. Insets show higher magnification of the merged image to highlight colocalization between GFAP and TREK-2 channels in astrocytes. (TIF) [file pone.0125195.s001.tif]
